# Silicon differentially protects PSII and PSI photochemistry and preserves fatty acid metabolism in salt‐stressed *Sorghum bicolor* (L.)

**DOI:** 10.1002/jsfa.71027

**Published:** 2026-09-07

**Authors:** Farah Bounaouara, Rabaa Hidri, Mohammed Falouti, Salma Nait Mohamed, Mokded Rabhi, Ahmed Hichem Hamzaoui, Issam Nouairi, Nabil Ben Youssef, Cécile Cabassa, Arnould Savouré, Chedly Abdelly, Inès Slama, Walid Zorrig

**Affiliations:** ^1^ Laboratory of Extremophile Plants Centre of Biotechnology of Borj‐Cedria Hammam‐Lif Tunisia; ^2^ Laboratory of Olive Biotechnology Centre of Biotechnology of Borj‐Cedria Hammam‐Lif Tunisia; ^3^ Department of Plant Production, College of Agriculture and Food Qassim University Buraydah Saudi Arabia; ^4^ Laboratory of Valorization of Useful Materials National Centre for Research in Materials Sciences, Technopole of Borj Cedria Soliman Tunisia; ^5^ Laboratory of Legumes and Sustainable Agrosystems Centre of Biotechnology of Borj‐Cedria Hammam‐Lif Tunisia; ^6^ Sorbonne Université, UPEC, CNRS, IRD, INRAE, Institute of Ecology and Environmental Science of Paris (iEES) Paris France

**Keywords:** fatty acids, photosystems, salinity, secondary structure, silicon, sorghum

## Abstract

**BACKGROUND:**

Silicon (Si) has been demonstrated to be an important element to enhance crop tolerance against salinity. Nevertheless, mechanisms underlying Si's mitigation of salt stress remains insufficiently explored and understood.

This study aims to provide new insights into Si's impact on salt‐stressed *Sorghum bicolor* (L.) supplied or not with 0.5 mmol L^−1^ of sodium silicate (Na_2_SiO_3_) by combining the assessment of photosystems performance, fatty acid metabolism as linked to membrane integrity, and molecule's chemical structure stability.

**RESULTS:**

Relative to sodium chloride (NaCl) treatment alone, Si supply enhanced biomass production; increased chlorophyll *a*, chlorophyll *b*, and carotenoid contents by 35%, 26%, and 61%, respectively (*P* < 0.01); and increased proline and total soluble sugar contents (*P* < 0.05).

Notably, results shed new light into Si‐optimized photochemical balance between photosystems by further enhancing photosystem II (PSII) photochemical yield (Y(II)), electron transport rate (ETR(II)) and diminished non‐photochemical energy dissipation processes (Y(NPQ) and Y(NO)), while recovering photosystem I (PSI) photochemical yield (Y(I)) and electron transport rate (ETR(I)), primarily via the alleviation of the donor‐side limitation (decrease in Y(ND)). Moreover, Si supplementation preserved membrane integrity, reducing malondialdehyde (MDA) levels by 15% (*P* < 0.01), as demonstrated by enhanced linoleic acid (C18:2) and linolenic acid (C18:3) contents (*P* < 0.05). Further, results showed a previously unreported observation of Si's protective effect on the chemical structure of molecules by suppressing the induced salt modifications.

**CONCLUSION:**

In summary, this study broadens our understanding of Si's enhanced salt tolerance, showing improved balance of photosystem's performance, maintaining membrane stability and fatty acid metabolism, and preserving the integrity of chemical secondary structures. © 2026 The Author(s). *Journal of the Science of Food and Agriculture* published by John Wiley & Sons Ltd on behalf of Society of Chemical Industry.

## INTRODUCTION

Global food security is an ongoing and evolving challenge due to the impacts of climate change. Extreme weather fluctuations, including unexpected increases in temperature and precipitation, and particularly the expansion of saline soils, are pushing crops beyond their adaptive ability, highlighting the urgent need for effective mitigation strategies.^1^ Globally, soil salinity has rendered approximately 6% of productive land‐equivalent to 800 million hectares‐uncultivable.^2^ Salinity impairs crop growth and yield by inducing ionic imbalances that cause nutritional stress, hyperosmotic stress that limits water uptake, and oxidative damage resulting from the over production of reactive oxygen species (ROS). These ROS disrupt normal metabolism by oxidatively modifying macromolecules, including lipids, proteins, and nucleic acids, thereby affecting multiple vital physiological processes.^3^, ^4^ For instance, photosynthesis is a very important physiological process that is closely associated with optimal plant growth.^5^ Consequently, the alteration in photosynthesis under salt stress is directly linked to declines in crop yield. In fact, salt accumulation in photosynthetically active leaf mesophyll cells inhibits key processes of photosynthesis, including the activity of Calvin cycle enzymes, the biosynthesis of photosynthetic pigments, and the efficiency and structural integrity of photosystems and thylakoid membranes.^6^

In addition, salt accumulation also disrupts the stability of a cell's plasma membrane.^7^ In fact, biological membranes are predominantly composed of fatty acids (FAs).^8^ FAs are classified as monounsaturated fatty acids (MUFAs, mainly oleic acid, C18:1), polyunsaturated fatty acids (PUFAs, primarily linoleic acid, C18:2, and linolenic acid, C18:3), and saturated fatty acids (SFAs, including palmitic acid, C16:0, and stearic acid, C18:0) and are primarily synthesised in chloroplasts and plastids.^9^ These FAs are key components of plasma membranes, including chloroplast and thylakoid membranes, and play a central role in maintaining membrane structure and function by modulating its fluidity and permeability.^10^, ^11^, ^12^ Under salt stress, plants undergo FA remodelling, which is likely responsible for changes in membrane properties.^13^ Membrane permeability is a critical determinant of salt tolerance in plants, and alterations in this feature are closely linked to variations in FA composition and the degree of unsaturation, which are considered indicators of stress tolerance or sensitivity.^14^, ^15^ Notably, modifications in membrane fluidity create an environment conducive to the functioning of key integral proteins, including the photosynthetic machinery, under stress conditions.^16^ Nevertheless, and despite its crucial importance for plant development, FA metabolism has been largely overlooked in studies investigating plant responses to abiotic stresses, particularly salinity, with most research focusing on cold and heat stress.^9^, ^17^ In this context, understanding plant photosynthetic and FA metabolism in response to salt stress is essential for identifying tolerance traits in species and also for elucidating the mechanisms underlying strategies aimed at improving crop salt tolerance. Such insights are a primary priority for addressing global food security in the context of an increasing world population.^18^

Silicon (Si) treatment has been recognized as one of the effective approaches for enhancing plant tolerance and mitigating the adverse effects of abiotic stressors, including salinity.^19^, ^20^, ^21^, ^22^, ^23^, ^24^, ^25^ In fact, Si, one of the most abundant elements in the Earth's crust, accounts for approximately 28% of the lithosphere and has recently been classified as a ‘quasi‐essential’ element by the International Plant Nutrition Institute.^26^ One key mechanism underlying Si‐mediated enhancement of crop tolerance is the reduction of toxic salt ion (sodium ion, Na^+^, and chloride ion, Cl^−^) uptake. This effect has been widely reported in several species, including alfalfa, bean, wheat, and sorghum.^27^, ^28^, ^29^, ^30^, ^31^ For example, Gong *et al*.^32^ demonstrated that Si deposition in the root endodermis restricts Na^+^ transport via the ‘transpirational bypass’ pathway from roots to stems in rice. Moreover, polymerization of Si within the apoplast (biosilicification) forms an amorphous silica barrier that reinforces root cell structure and prevents Na^+^ penetration into the symplast or transpiration stream.^33^ Nevertheless, the magnitude of Si's beneficial effects under high salinity levels varies considerably among plant species and is largely determined by the plant's capacity to uptake Si and its inherent sensitivity to salt stress.^34^ More critically, knowledge regarding Si's effect on key metabolic pathways, precise photosynthesis, together with FA metabolism and membrane integrity, remains poorly explored and lacks a clear explanation.

Accordingly, the present study aims to uncover the physiological and biochemical response of salt‐stressed *Sorghum bicolor* plants to Si application. More precisely, this study focused on unveiling Si's potential protective effect, combining the assessment of the photochemical efficiency of photosystems (photosystem II, PSII, and photosystem I, PSI), leaf membrane stability as linked to FA metabolism, and osmotic adjustment. Adding to this, Si's effect on the molecular structural properties of *S. bicolor* leaves was examined to investigate whether Si also exhibits a restoring or enhancing effect on the possible molecular structural modifications induced by salt stress. Literature data regarding this aspect is very limited.

Combining all aspects of photosynthesis, membrane integrity, FA metabolism and molecule's structural stability, this study tests the hypothesis that Si improves the physiological and biochemical equilibrium of *S. bicolor* against salt stress, improving its growth and yield.

## MATERIALS AND METHODS

### Plant material and growth conditions

Seeds of *S. bicolor* were first put in Petri dishes to germinate and placed in an incubator at a temperature of 25°C and in obscure conditions. After 6 days of germination, *S. bicolor* seedlings were transferred to a glasshouse at the Centre of Biotechnology of Borj‐Cedria (northeast Tunisia; 36°42′32.9″ N, 10°25′40.9″ E) and transplanted into a bucket of 5 L (eight seedlings per bucket) filled with Hewitt's nutrient solution,^35^ which was continuously aerated using aquarium pumps. Experimental conditions were maintained at temperatures of 23–25°C, relative humidity of 60–80%, and natural sunlight with a photoperiod of approximately 16 h of continuous light intensities ranging from 400 to 1200 μmol photons m, followed by 8 h of uninterrupted darkness.

Following 23 days of acclimatisation, plants were assigned to one of three treatments: (i) control, (ii) salt stress (200 mmol L^−1^ sodium chloride (NaCl)), and (iii) salt stress supplemented with 0.5 mmol L^−1^ of Si in the form of sodium silicate (Na_2_SiO_3_; Sigma‐Aldrich, St Louis, MO, USA). Treatments were applied for 15 days.

The choice of 200 mmol L^−1^ NaCl was based on a previous experiment showing that *S. bicolor* showed tolerance to salt stress in response to 150 mmol L^−1^ NaCl (data not shown). The applied concentration of Si (0.5 mmol L^−1^) was also concluded based on literature and on a preliminary experiment (data not shown) proving that 0.5 mmol L^−1^ of Si was optimum to study its beneficial effect under salt stress. In addition, the experiment showed that supplementation with 0.5 mmol L^−1^ of Na_2_SiO_3_ under control conditions (0 mmol L^−1^ NaCl) did not affect plant growth, which is consistent with previous reports suggesting that Si confers minimal benefits under non‐stress conditions.^36^ Accordingly, this study exclusively focused on studying Si's application effect only in salt‐stressed *S. bicolor* plants. Consequently, the experiment retained three cultural conditions, including control, NaCl, and NaCl + Si. Further, other forms of Si (e.g., potassium silicate (K_2_SiO_3_) or calcium silicate (CaSiO_3_)) were avoided in this study to distinguish the beneficial effect of Si from other elements like potassium ion (K^+^) or calcium ion (Ca^2+^). Accordingly, the format Na_2_SiO_3_ was used, taking into account that the Na^+^ concentration derived from Na_2_SiO_3_ is negligible compared to the Na^+^ concentration added from NaCl (200 mmol L^−1^).

### Harvest and determination of growth parameters

At harvest, root and shoot lengths, as well as the number of leaves and tillers, were measured. Leaf and root samples were subsequently oven‐dried at 50 °C for 7 days to determine dry weight.

The salinity tolerance index (STI) was calculated using the following formula:

$$\mathrm{STI}=\left(\mathrm{TDW}1/\text{TDW2}\right)\times 100$$

where TDW1 represents the total dry weight (in milligrams) of salt‐stressed *S. bicolor* grown in the absence or presence of 0.5 mmol L^−1^ Si (Na_2_SiO_3_), and TDW2 represents the total dry weight (in milligrams) of control *S. bicolor* plants.

### Photosynthetic pigment concentrations

Fresh leaf discs (200 mg) were homogenized in 7 mL of 80% (*v/v*) acetone and incubated in the dark at 4 °C. After 7 days, the absorbance of the extracts was measured at 470, 646, and 663 nm using an ultraviolet (UV)‐visible spectrophotometer (Specord 210 Plus; Analytik Jena, Jena, Germany). The contents of chlorophyll *a* (Chl. *a*), chlorophyll *b* (Chl. *b*), total chlorophyll (Chl. *a* + Chl. *b*), and carotenoid (Car.) were calculated following the method of Lichtenthaler.^37^

### Silicon content

Silicon content in *S. bicolor* roots and leaves was determined using a colorimetric method, as described by Haddad *et al*.^38^ Dried and ground root and leaf tissues (10 mg) were homogenised with 300 μL of 50% sodium hydroxide (NaOH) and autoclaved at 121°C for 20 min. The volume was then adjusted to 500 μL with double‐distilled water (ddH_2_O). After centrifugation, 160 μL of the supernatant was transferred to a 2 mL tube containing 1.2 mL of 20% acetic acid. Subsequently, 400 μL of ammonium molybdate solution (43.7 mmol L^−1^, pH 7) was added and mixed thoroughly. After 5 min, 200 μL of 20% tartaric acid and 40 μL of reducing solution‐prepared by mixing solution A (2 g Na_2_SO_3_ and 0.4 g 1‐amino‐2‐naphthol‐4‐sulfonic acid in 25 mL water) and solution B (25 g NaHSO_3_ in 200 mL ddH_2_O, adjusted to 250 mL with ddH_2_O)‐were added, and the mixture was allowed to rest for 30 min. Absorbance was measured at 650 nm, and Si content was quantified using a calibration curve prepared with a Na_2_SiO_3_ solution.

The silicon translocation factor from roots to shoots was calculated as follows:

SiTranslocation Factor=ShootSicontent/rootSicontent.

### Total soluble sugar content

Total soluble sugar (TSS) content was determined following the method of DuBois *et al*.^39^ Fresh leaf and root samples were first grounded in liquid nitrogen, and 50 mg of grounded material was homogenized in 1.5 mL of 80% ethanol. After centrifugation, 0.5 mL of the supernatant was mixed with 1 mL of 5% phenol and 2.5 mL of concentrated sulphuric acid (H_2_SO_4_), and the mixture was allowed to cool for 30 min. Absorbance was measured at 485 nm, and TSS content was quantified using a calibration curve prepared with a glucose standard solution.

### Proline content

Proline content was determined following the method of Bates *et al*.^40^ Briefly, 50 mg of crushed fresh leaf and root tissue was mixed with 1 mL of 3% sulfosalicylic acid. After centrifugation, 400 μL of the supernatant was combined with 400 μL of glacial acetic acid and 400 μL of ninhydrin reagent (2.5% *w/v* of ninhydrin dissolved in 60% glacial acetic acid and 40% phosphoric acid 6 mol L^−1^), and the mixture was incubated at 100 °C for 1 h. The reaction mixture was then cooled on ice, and 800 μL of toluene was added. Absorbance of the toluene phase was measured at 520 nm, and proline content was quantified using a calibration curve prepared with a l‐proline standard solution.

### Lipid peroxidation assessment

Leaf membrane stability was evaluated through lipid peroxidation by quantifying malondialdehyde (MDA) content, following the method of Heath and Packer.^41^ Dry leaf and root tissues (100 mg) were homogenized in 2 mL of extraction solution (20% trichloroacetic acid (TCA) and 0.5% thiobarbituric acid (TBA) using a mortar and pestle in an ice bath. The homogenate was incubated at 95 °C for 30 min, and the reaction was stopped in a cold‐water bath. After centrifugation at 11 000 rpm for 10 min at 4 °C, the absorbance of the supernatant was measured at 532 and 600 nm using a UV‐visible spectrophotometer (Specord 210 Plus). Content of MDA was expressed as μmol g^−1^ dry weight.

### Photosystem II and photosystem I activities

PSII and PSI activities were measured using a Dual‐PAM‐100 system (Heinz Walz, Effeltrich, Germany) on *S. bicolor* leaves that had been dark‐adapted for 30 min prior to measurement, following the procedure described by Klughammer and Schreiber.^42^, ^43^

For PSII, dark‐adapted leaves were subjected to a rapid light‐response curve at photosynthetic active radiation (PAR) levels of 0, 6, 12, 21, 56, 107, 146, 257, 412, 652, and 1017 μmol photons m^−2^ s^−1^. The measured parameters were photochemical yield (Y(II)), diminished non‐photochemical energy dissipation processes (Y(NPQ) and Y(NO)), the apparent electron transport rate ETR(II), and the photochemical quenching coefficients qP and qL, which estimate the fraction of open PSII reaction centres under the ‘puddle’ and ‘lake’ antenna‐connectivity models, respectively. Each light step lasted at least 30 s; therefore, the resulting values represent rapid‐light‐curve responses rather than fully stabilized steady‐state responses.

PSI activity was evaluated using the Dual‐PAM‐100 P700 dual‐wavelength emitter‐detector units (830 and 875 nm), allowing assessment of PSI photochemical efficiency.^43^ Parameters measured included the quantum yield of photochemical energy conversion in PSI (Y(I)), the quantum yield of non‐photochemical energy dissipation limited by the acceptor side (Y(NA)), the quantum yield of non‐photochemical energy dissipation limited by the donor side (Y(ND)), the electron transport rate (ETR(I)), and the oxidation state of PSI (P700^+^). Saturating pulses were applied during the induction curve to transiently reduce the photo‐oxidizable P700 pool and determine the maximal P700 signals required to calculate Y(I), Y(ND), and Y(NA).

### Lipid extraction

Lipid extraction was performed following the method of Folch *et al*.^44^ as adapted by Koberg *et al*.^45^ Leaf samples were first lyophilized and grounded into a fine powder using a mortar and pestle. Next, 1 g of powdered tissue was mixed with a chloroform–methanol solution (2:1, *v/v*) and vortexed thoroughly. Subsequently, 2 mL of 0.9% NaCl solution was added, and the mixture was subjected to ultrasonic treatment to ensure complete extraction. After centrifugation, the lower chloroform phase was collected and evaporated using a rotary evaporator to obtain the dry lipid residue.

### Fatty acid methyl esters preparation and gas chromatography‐flame ionization detection

Fatty acid methyl esters (FAMEs) were prepared from the dry lipid residues of *S. bicolor* leaves via cold alkaline transesterification. Briefly, 200 mg of dry lipid residue was dissolved in 3 mL of hexane and vigorously mixed with 0.4 mL of 2 M methanolic potassium hydroxide (KOH).

FAMEs were analysed using a Hewlett‐Packard chromatograph (HP 4890 D; Hewlett‐Packard Company, Wilmington, DE, USA) equipped with a flame ionization detector (FID) and a silica capillary column (30 m × 0.25 mm diameter × 0.25 μm film thickness; Agilent, Wilmington, DE, USA). An injection volume of 1 μL was used. The injector, detector, and oven temperatures were maintained at 220 °C, 250 °C, and 210 °C, respectively. FAs were identified based on their retention times.

The double bond index (DBI), representing the weighted degree of unsaturation of FAs, was calculated as follows^46^:

$$\mathrm{DBI}=\left(C16:1\right)+\left(C18:1\right)+\left[2\times \left(C18:2\right)\right]+\left[3\times \left(C18:3\right)\right]$$

### Fourier‐transform infrared spectroscopy

The grounded dry leaf tissue of *S. bicolor* was analysed using attenuated total reflection‐Fourier transform infrared (ATR‐FTIR) spectroscopy, in which infrared light is reflected by a crystal and interacts with the sample to identify chemical bonds. Infrared spectra, expressed as absorbance, were recorded using a Spectrum II spectrometer (PerkinElmer, Liantrisant, UK) over a range of 500–4000 cm^−1^. Spectral data were processed using Origin software, version 2024.

### Statistical analysis

All statistical analyses were performed using XLSTAT software, version 2014 (www.xlstat.com). The analyses included: (i) comparison of means between two independent sample groups using Student's *t*‐test at three significance levels (*P* < 0.05, *P* < 0.01, and *P* < 0.001); (ii) Duncan's Multiple Range Test to compare means with a 95% confidence level; (iii) Pearson correlation analysis; and (iv) principal component analysis (PCA), with variables centred on their means and normalized to a standard deviation of 1.

## RESULTS

### Influence of silicon on the morphological and phenotypic characteristics of sorghum

Exposure of *S. bicolor* to salinity stress (200 mmol L^−1^ NaCl) resulted in reduced plant size and growth compared to the control (Fig. 1). Leaves exhibited signs of necrosis and were narrower and less developed.

**Figure 1:**
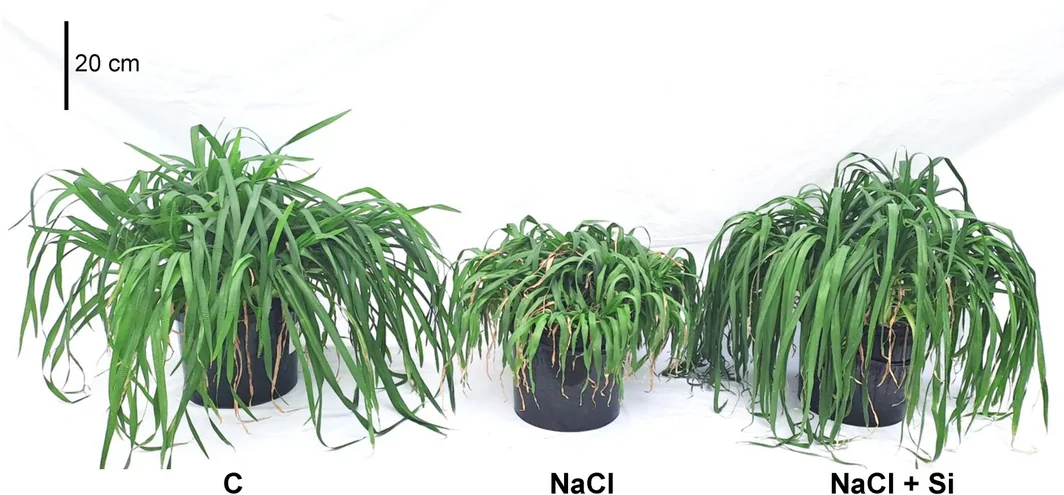
Morphological appearance of control (C) and salt‐treated (200 mmol L^−1^ NaCl) *Sorghum bicolor* plants with or without silicon (Si) supplementation (0.5 mmol L^−1^ Na_2_SiO_3_) after 15 days of treatment. The morphological responses shown in this figure were consistent across three independent experiments.

In contrast, *S. bicolor* plants grown with Si supplementation in the saline medium showed a marked improvement in growth and vigour compared to salt‐stressed plants, with more fully developed leaves, underscoring the beneficial effect of Si on plant productivity under salt stress.

### Effect of silicon on sorghum's growth and yield

Plants grown under salt stress exhibited a reduction in root, shoot, and total plant biomass by 56%, 41%, and 54%, respectively (Fig. 2). However, the addition of Si to salt‐stressed *S. bicolor* plants restored root, shoot, and total dry weight, with no significant differences observed between the control and NaCl + Si treatments.

**Figure 2:**
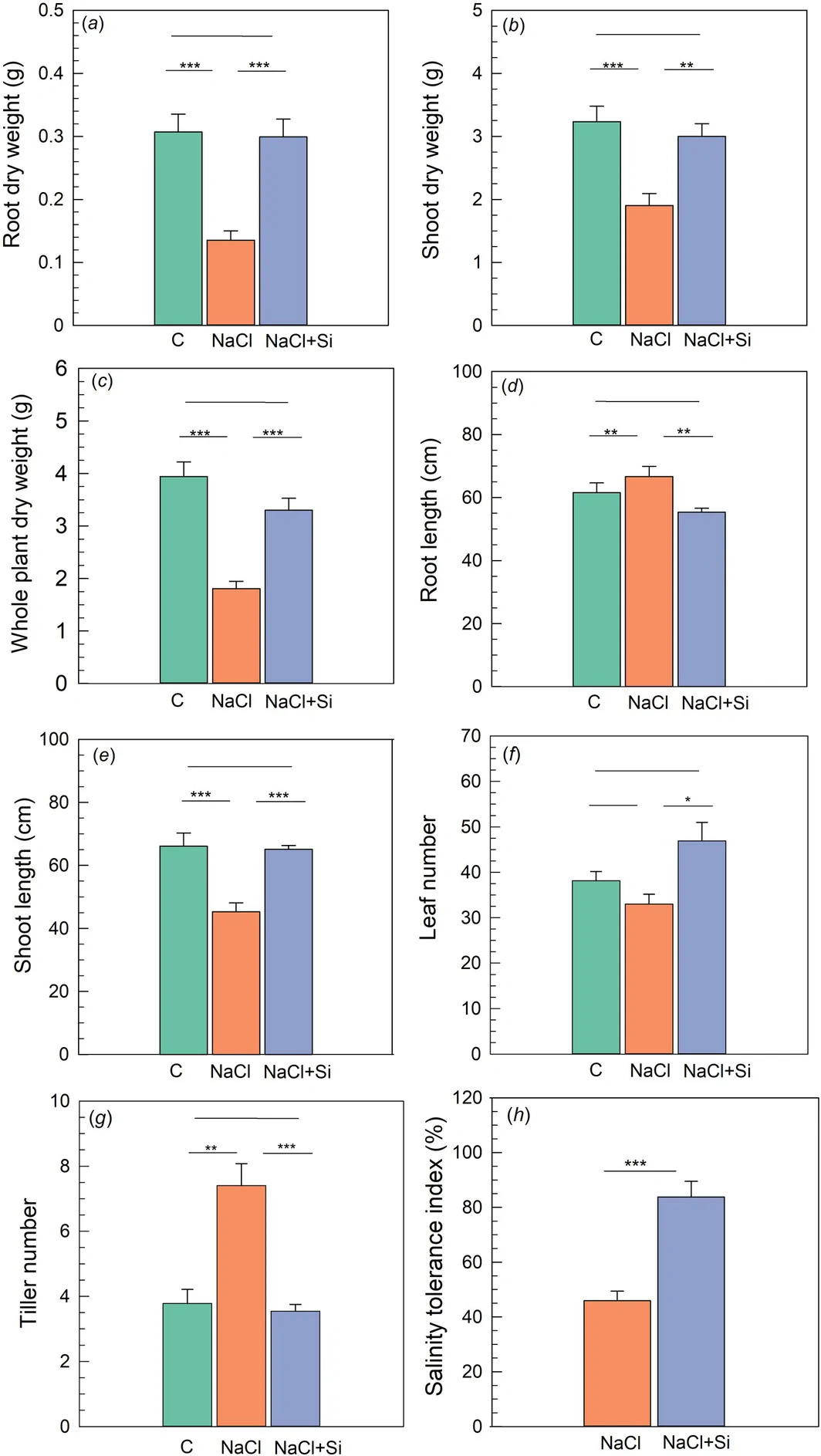
Growth parameters of control (C), salt‐treated (200 mmol L^−1^ NaCl) *Sorghum bicolor* plants with or without silicon (Si) supplementation (0.5 mmol L^−1^ Na_2_SiO_3_) after 15 days of treatment: (a) root dry weight, (b) shoot dry weight, (c) whole plant dry weight, (d) root length, (e) shoot length, (f) leaf number, (g) tiller number, and (h) salinity tolerance index. Values represent mean ± standard error of seven replicates. Asterisks indicate statistically significant differences according to Student's t‐test (*P < 0.05; **P < 0.01; ***P < 0.001). The absence of asterisks indicates no significant differences.

Furthermore, the results revealed that salt‐stressed *S. bicolor* plants displayed an increase in root length but a decrease in shoot length (Fig. 2(d),(e)). With Si supplementation, both root and shoot lengths were significantly restored to values statistically comparable to those of the control plants.

### Regulation of photosynthetic pigment concentrations

Leaves of *S. bicolor* plants exposed to 200 mmol L^−1^ NaCl for 15 days exhibited a significant decrease in Chl. *a*, total chlorophyll (Chl. *a* + Chl. *b*), and Car. contents, whereas Chl. *b* content remained unaffected (Fig. 3). Silicon supplementation led to an increase in Chl. *a*, total chlorophyll, and Car. contents by 35%, 32%, and 61%, respectively, compared to salt‐stressed plants. Additionally, Chl. *b* content increased by 26% with Si addition under salt stress.

**Figure 3:**
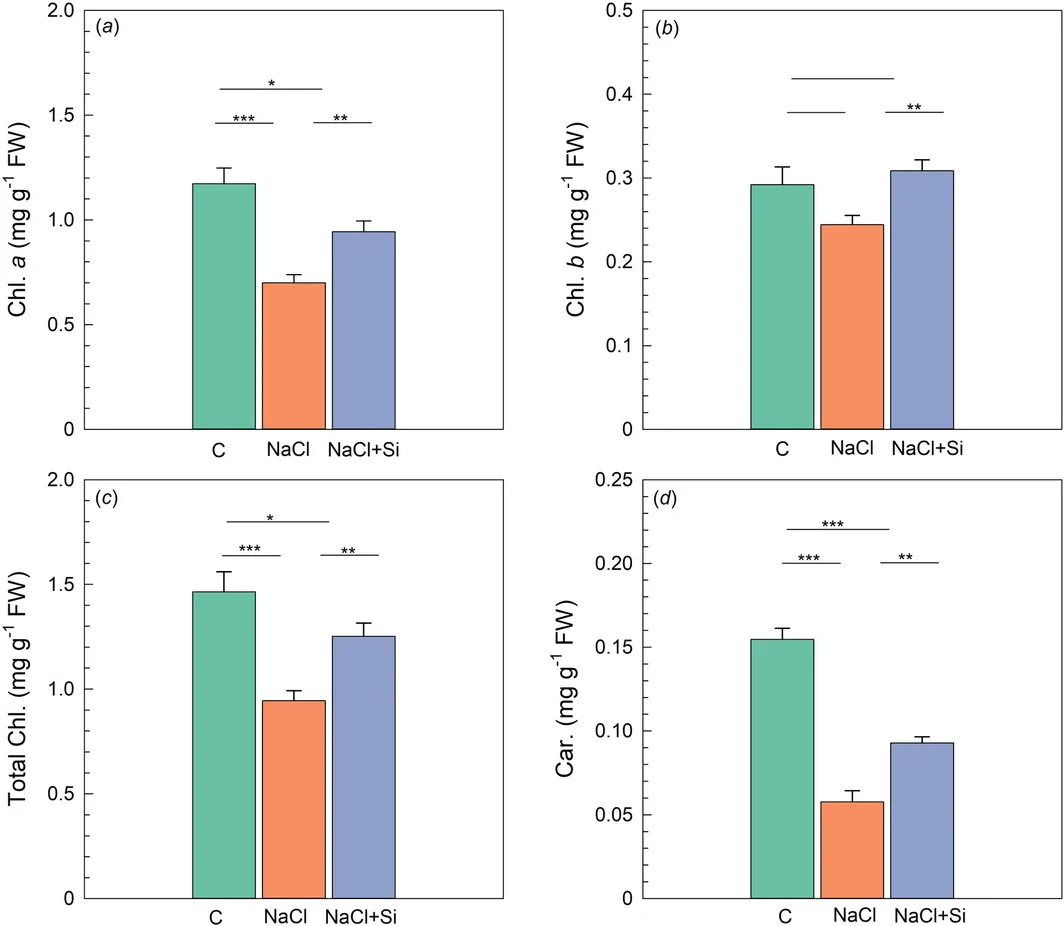
Photosynthetic pigment contents (mg g^−1^ fresh weight (FW)) in leaves of control (C), salt‐treated (200 mmol L^−1^ NaCl), and salt‐treated *Sorghum bicolor* plants supplemented with silicon (Si) (0.5 mmol L^−1^ Na_2_SiO_3_): (a) chlorophyll *a* (Chl. *a*), (b) chlorophyll *b* (Chl. *b*), (c) total chlorophyll (Total Chl.), and (d) carotenoid (Car.). Values represent mean ± standard error of seven replicates. Asterisks denote statistically significant differences (*P < 0.05; **P < 0.01; ***P < 0.001). The absence of asterisks indicates no significant differences.

### Silicon tissue content and accumulation

Under salt stress, the addition of 0.5 mmol L^−1^ Si to the culture medium resulted in a significant accumulation of Si, primarily in the leaves of *S. bicolor*, reaching 1.69% of leaves dry weight after 15 days of treatment, approximately ten times higher than in the roots. Moreover, a markedly high Si translocation factor from roots to leaves was observed (Fig. 4).

**Figure 4:**
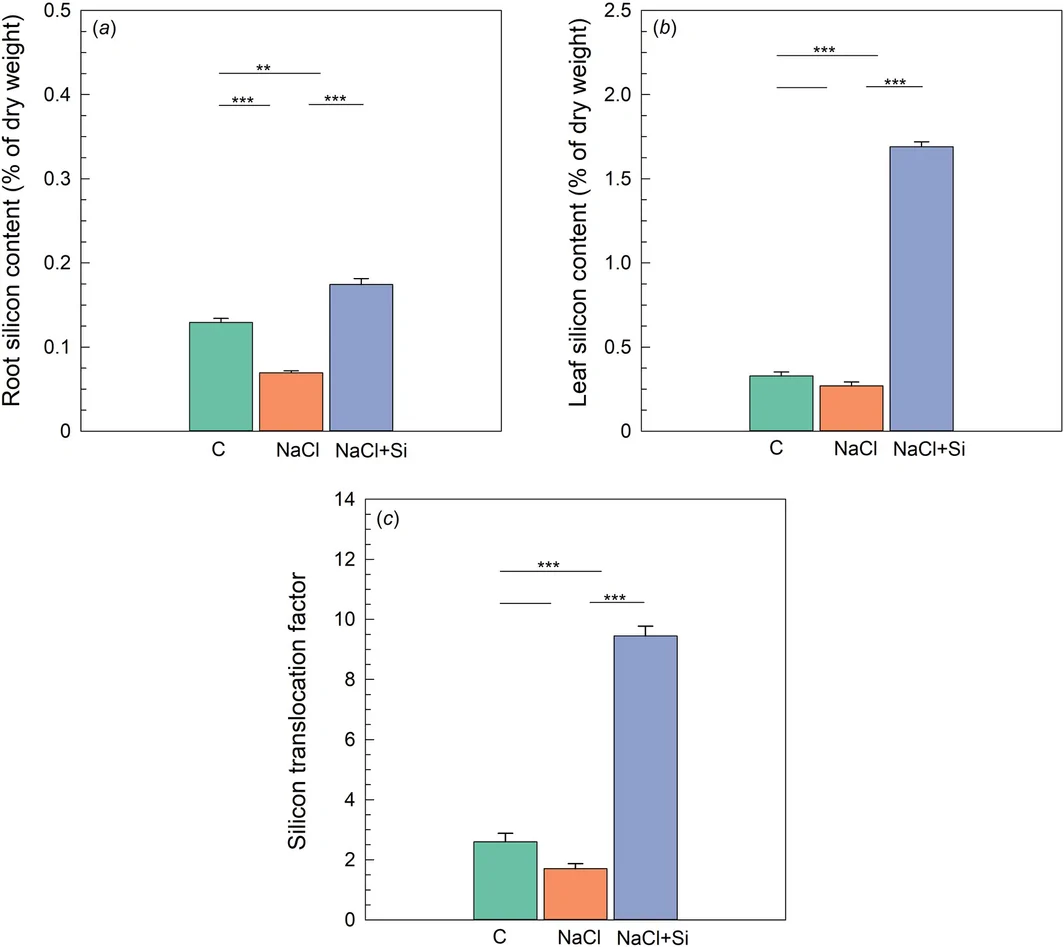
Root and leaf silicon (Si) contents (a, b) and Si translocation factor from roots to leaves (c) in control (C), salt‐treated (200 mmol L^−1^ NaCl), and salt‐treated *Sorghum bicolor* plants with Si supplementation (0.5 mmol L^−1^ Na_2_SiO_3_). Values represent mean ± standard error of seven replicates. Asterisks indicate statistically significant differences (*P < 0.05; **P < 0.01; ***P < 0.001). The absence of asterisks indicates no significant differences.

### Silicon effect on osmolytes accumulation

Under salt stress, proline and TSS contents increased significantly in both roots and leaves compared to the control (Fig. 5). Proline accumulation was particularly pronounced, with levels in roots and leaves reaching nine‐ and six‐fold higher, respectively, than in control plants. Similarly, TSS contents increased five‐ and six‐fold in roots and leaves, respectively, under salt stress.

**Figure 5:**
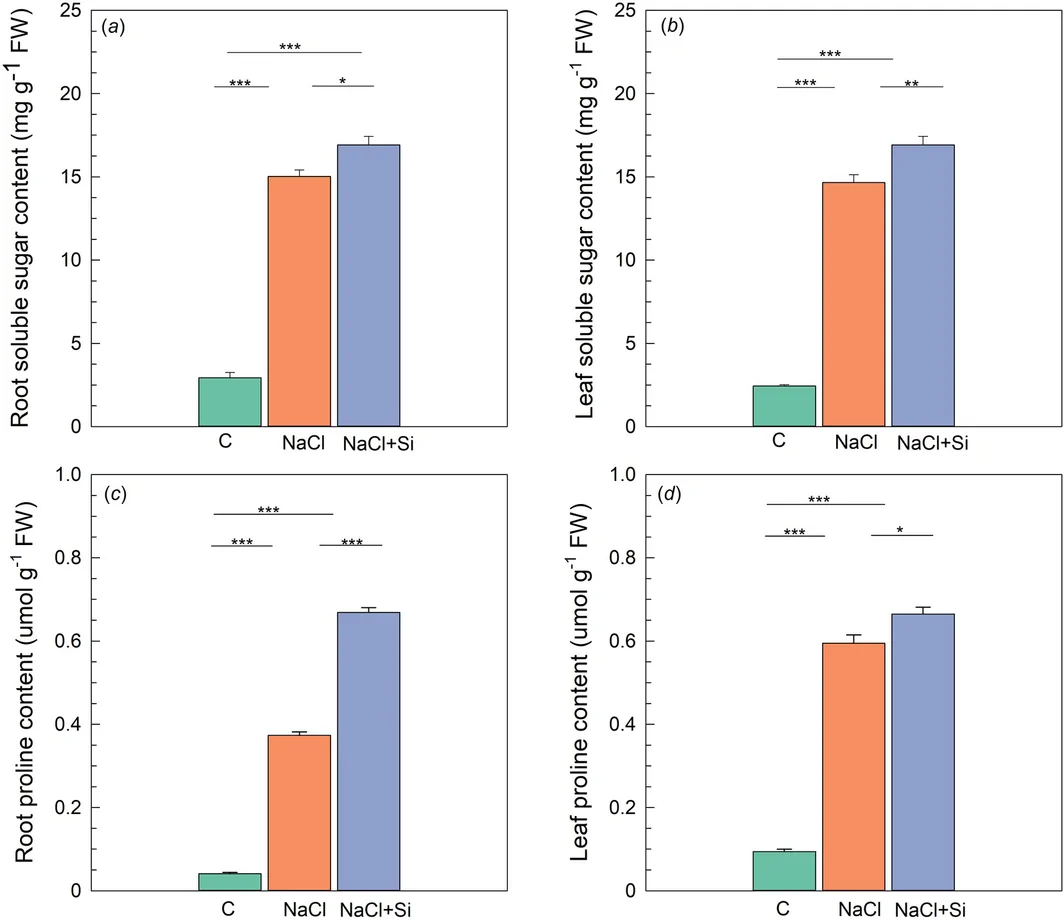
Osmotic adjustment in control (C), salt‐treated (200 mmol L^−1^ NaCl), and silicon (Si)‐supplemented (0.5 mmol L^−1^ Na_2_SiO_3_) *Sorghum bicolor* plants: (a, b) total soluble sugar content in roots and leaves, (c, d) proline content in roots and leaves. Values represent mean ± standard error of seven replicates. Asterisks indicate statistically significant differences (*P < 0.05; **P < 0.01; ***P < 0.001). The absence of asterisks indicates no significant differences.

Silicon supplementation of the salt‐stressed medium further enhanced proline accumulation, with increases of 80% in roots and 13% in leaves. TSS content was also elevated in both organs with Si addition, showing significant increases of 9% in roots and 15% in leaves of salt‐stressed *S. bicolor* plants.

### Silicon's photoprotective effect towards photosystem II and photosystem I

Analysis of the functional efficiency of PSII and PSI revealed a decline in photochemical energy conversion yields of PSII (Y(II)) and PSI (Y(I)) under progressively increasing PAR intensities (6–1017 μmol photons m^−2^ s^−1^) across all growth conditions (control, NaCl, and NaCl + Si) (Figs 6 and 7). Under salt stress, the two photosystems exhibited distinct responses: compared to the control, PSII showed an increase in Y(II), whereas PSI displayed a decrease in Y(I). Silicon supplementation further enhanced Y(II), producing values higher than both control and NaCl conditions, and restored Y(I) to control levels.

**Figure 6:**
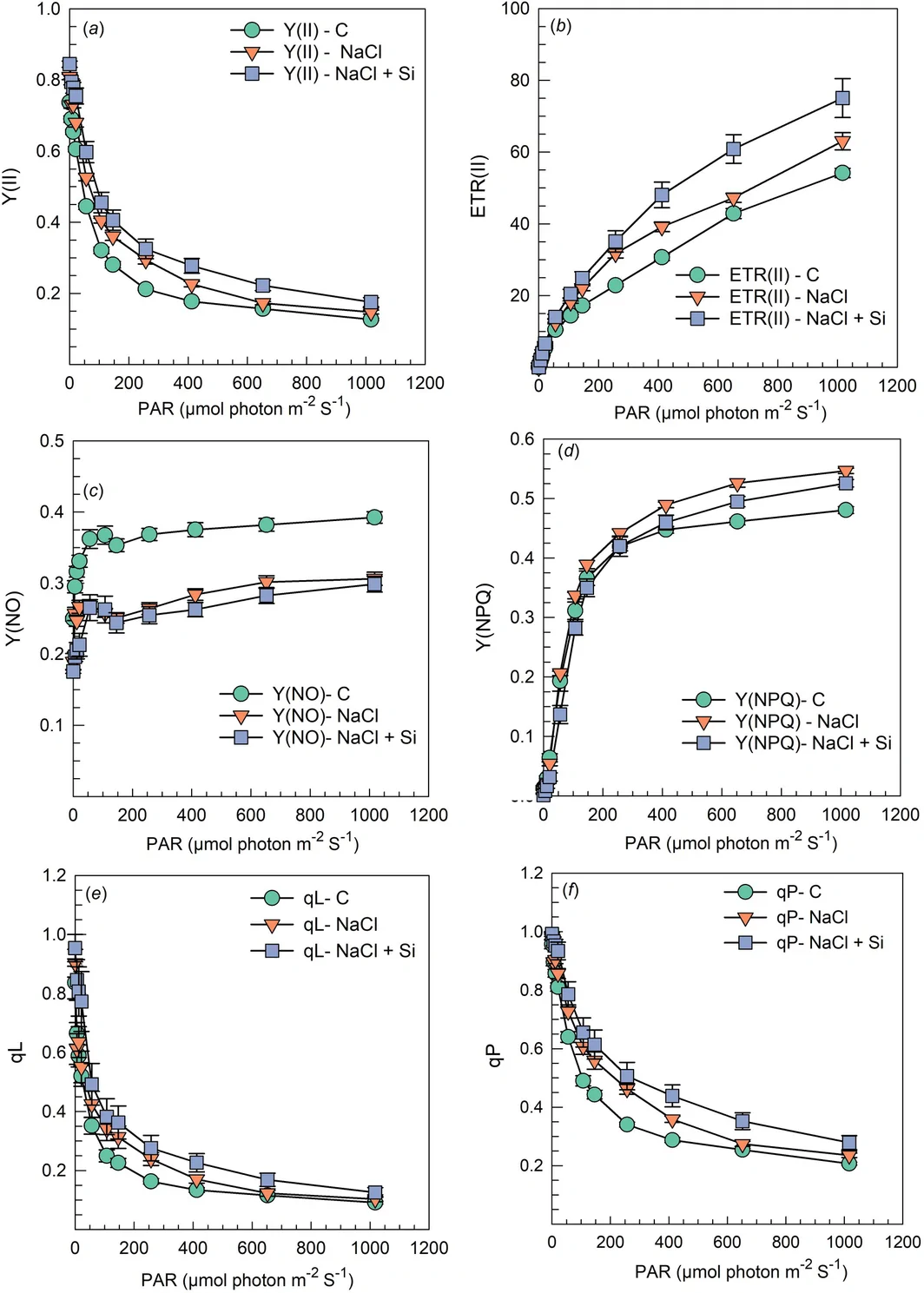
Effect of silicon (Si) on photosystem II (PSII) photochemical activity in leaves of control (C), salt‐treated (NaCl), and salt‐treated *Sorghum bicolor* plants supplemented with 0.5 mmol L^−1^ Na_2_SiO_3_ (NaCl + Si), after a minimum exposure of 30 s at each light intensity (photosynthetic active radiation, PAR): (a) Y(II) – quantum yield of photochemical energy conversion in PSII, (b) ETR(II) – electron transfer rate in PSII, (c) Y(NO) – quantum yield of non‐regulated non‐photochemical energy dissipation in PSII, (d) Y(NPQ) – quantum yield of regulated non‐photochemical energy dissipation in PSII, (e) qL – proportion of open PSII centres, (f) qP – fraction of photons absorbed by P680 used by open reaction centres. Values are mean ± standard error of five replicates.

**Figure 7:**
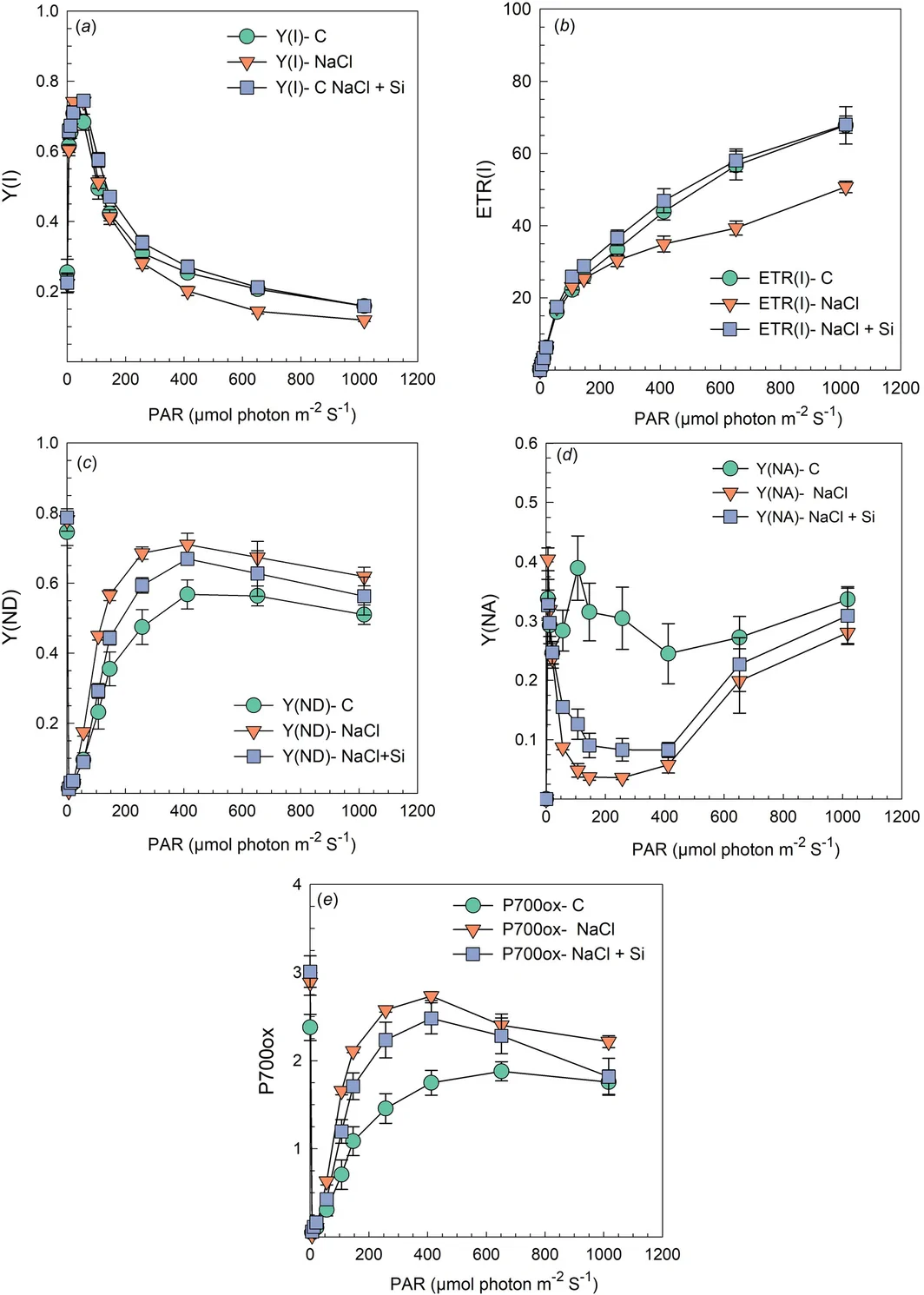
Effect of silicon (Si) on photosystem I (PSI) photochemical activity in light‐adapted leaves of control (C), salt‐treated (NaCl), and salt‐treated *Sorghum bicolor* plants supplemented with 0.5 mmol L^−1^ Na_2_SiO_3_ (NaCl + Si), after a minimum exposure of 30 s at each light intensity (photosynthetic active radiation, PAR): (a) Y(I) – quantum yield of photochemical energy conversion in PSI, (b) ETR(I) – electron transfer rate in PSI, (c) Y(ND) – quantum yield of non‐photochemical energy dissipation limited by the donor side in PSI, (d) Y(NA) – quantum yield of non‐photochemical energy dissipation limited by the acceptor side in PSI, (e) P700ox – oxidation state of PSI. Values are mean ± standard error of five replicates.

Regardless of growth condition, electron transport rates in PSII (ETR(II)) and PSI (ETR(I)) increased with rising PAR intensity. Consistent with the photochemical yields, ETR(II) was stimulated under salt stress, while ETR(I) declined relative to the control. With Si addition, ETR(II) was further enhanced, surpassing both control and NaCl treatments, whereas ETR(I) returned to control levels.

Furthermore, the decline in Y(II) was accompanied by increases in Y(NPQ) and Y(NO) as PAR intensity increased across all conditions. Salt‐stressed plants exhibited higher Y(NPQ) and lower Y(NO) compared to the control. Silicon supplementation slightly reduced Y(NPQ) toward control values and further suppressed Y(NO).

Salt stress increased both qP and qL, indicating a higher estimated fraction of open PSII reaction centres under the ‘puddle’ and ‘lake’ antenna‐connectivity models, respectively. This increase was more pronounced with Si supplementation, reflecting the trend observed for Y(II).

Additionally, under salt stress, the quantum yield of non‐photochemical energy dissipation limited by the acceptor side (Y(NA)) decreased below control levels, whereas the quantum yield limited by the donor side (Y(ND)) increased. The P700^+^ oxidation (P700ox) was also elevated under salt stress. Silicon supplementation partially restored these parameters, with Y(NA), Y(ND), and P700ox values approaching those of the control.

### Effect of silicon on cell's membrane stability

In *S. bicolor* plants exposed to 200 mmol L^−1^ NaCl, leaf MDA content increased by 36% compared to the control (Fig. 8). However, supplementation with Si in the salt‐stressed medium significantly mitigated this effect, resulting in a 15% reduction in MDA content relative to salt‐stressed leaves.

**Figure 8:**
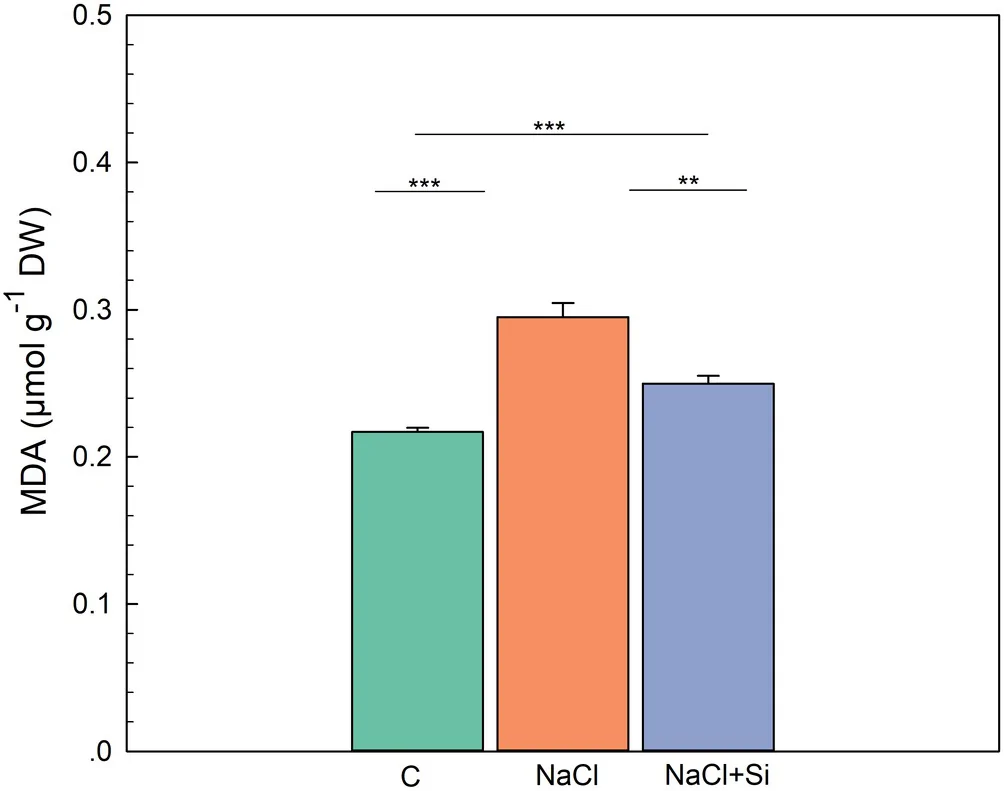
Leaf malondialdehyde (MDA) content in control (C), salt‐treated (200 mmol L^−1^ NaCl), and salt‐treated *Sorghum bicolor* plants supplemented with silicon (Si) (0.5 mmol L^−1^ Na_2_SiO_3_). Values represent mean ± standard error of seven replicates. Asterisks denote statistically significant differences (*P < 0.05; **P < 0.01; ***P < 0.001). The absence of asterisks indicates no significant differences.

### Silicon protective effect on leaf fatty acids (FAs) metabolism

The FA composition of *S. bicolor* leaves varied significantly with growth conditions (Table 1). In control leaves, the most abundant FAs were C18:3, C16:0, C18:2, palmitoleic acid (C16:1), C18:1, and C18:0. Under 200 mmol L^−1^ NaCl treatment, nearly all FAs showed a significant increase relative to the control, resulting in a reordering of abundance: C16:1, C16:0, C18:3, C18:1, C18:2, and C18:0 (Table 1). Despite the overall increase, C18:2 and C18:3 decreased significantly compared to the control, reducing total PUFAs and unsaturated fatty acids (UFAs), as well as the UFA/SFA ratio. The DBI of FAs decreased by 65.24% under salt stress relative to control.

**Table 1:** Fatty acid composition in leaves of control (C), salt‐treated (200 mmol L^−1^ NaCl), and salt‐treated *Sorghum bicolor* plants supplemented with 0.5 mmol L^−1^ Na_2_SiO_3_ (NaCl + Si), as determined by gas chromatography

	Fatty acid (%)		C	NaCl	NaCl + Si
Hexadecanoic acid	Palmitic acid	C16:0	11.28 b	33.57 a	11.89 b
9‐Hexadecanoic acid	Palmitoleic acid	C16:1	4.82 c	41.45 a	9.97 b
Octadecanoic acid	Stearic acid	C18:0	0.03 b	1.84 a	0.17 b
9‐Octadecenoic (ω‐9) acid	Oleic acid	C18:1	1.33 a	7.60 a	0.24 a
9.12‐Octadecadienoic (ω‐6) acid	Linoleic acid	C18:2	6.85 a	3.61 b	8.19 a
9.12.15‐Octadecatrienoic (ω‐3) acid	Linolenic acid	C18:3	75.65 a	10.14 b	68.79 a
Fatty acids biomarkers (%)		DBI	246.79 a	85.78 c	232.87 b
		SFA	11.30 b	35.41 a	12.06 b
		MUFA	6.15 b	47.07 a	10.13 b
		PUFA	82.50 a	13 c	76.98 b
		UFA	88.65 a	62.65 b	87.11 a
		UFA/SFA	7.85 a	1.78 c	7.23 b

*Note*: Values are means of three replicates. Different letters indicate statistically significant differences according to Duncan's test (*P* < 0.05). DBI, double bond index; SFA, saturated fatty acid; MUFA, monounsaturated fatty acid; PUFA, polyunsaturated fatty acid; UFA, unsaturated fatty acid; UFA/SFA, unsaturated fatty acid/saturated fatty acid ratio; NaCl, sodium chloride; Si, silicon; Na_2_SiO_3_, sodium silicate.

In leaves treated with NaCl + Si, the order of FA abundance closely resembled that of control leaves: C18:3, C16:0, C16:1, C18:2, C18:1, and C18:0 (Table 1). Consequently, the overall PUFA (76.98%), MUFA (10.13%), and SFA (12.06%) contents were significantly restored with Si supplementation. The DBI also increased significantly compared to salt‐stressed plants without Si.

### Fourier‐transform infrared analysis

FTIR spectra of control, NaCl‐, and NaCl + Si‐treated *S. bicolor* leaves exhibited notable differences across the identified peaks (Fig. 9). A total of 12 peaks were detected. The first band, around 3296 cm^−1^, corresponds to the stretching vibration of hydroxyl groups (–OH). Differences in absorbance among treatments suggest a higher –OH content in salt‐stressed leaves, which was reduced with Si supplementation, yielding a band closer to that of the control.

**Figure 9:**
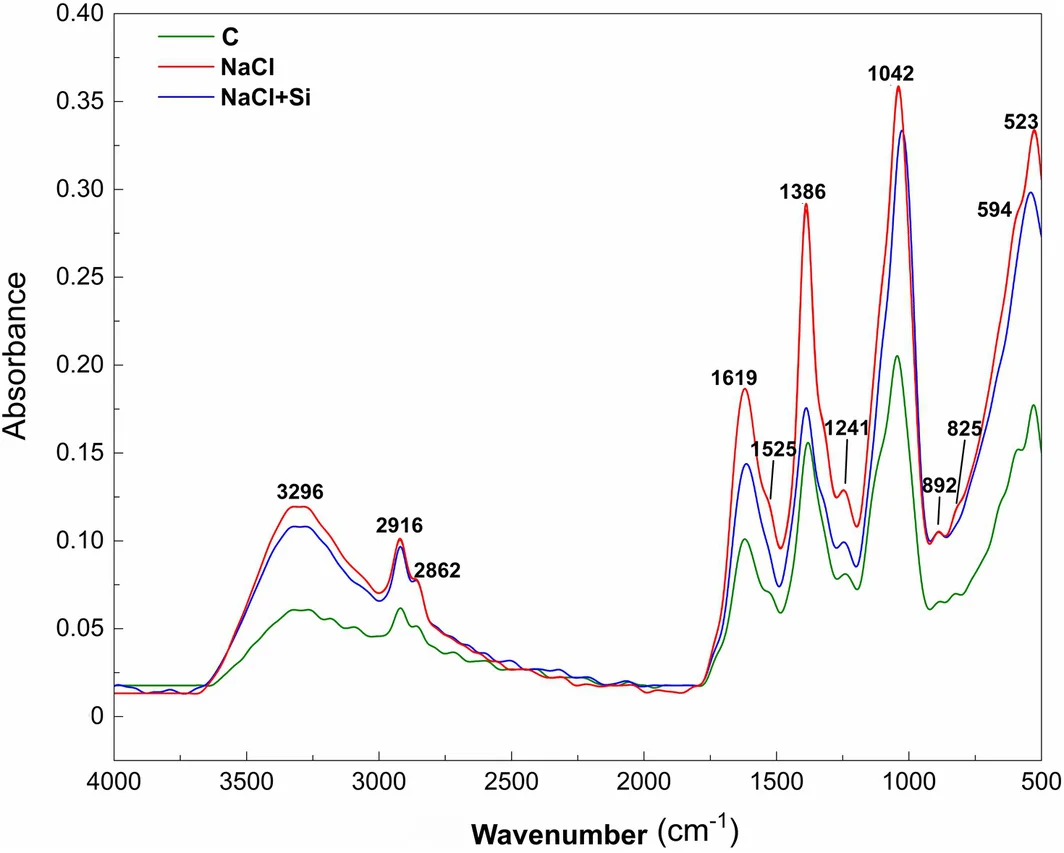
Fourier‐transform infrared (FTIR) spectra of control (C), salt‐treated (NaCl), and salt‐treated *Sorghum bicolor* leaves supplemented with silicon (NaCl + Si).

Two bands at approximately 2916 and 2862 cm^−1^, corresponding to C–H bond stretching vibrations, were observed in all treatments. The NaCl‐treated leaves exhibited the highest absorbance, while Si addition resulted in peaks overlapping with NaCl, indicating no effect of Si on C–H content.

A band at 1619 cm^−1^, attributed to C=C stretching vibrations, showed the highest absorbance under NaCl, indicative of the greater content of this group; NaCl + Si treatment exhibited intermediate values between control and NaCl, suggesting attenuation of accumulation with Si. A weak band at 1525 cm^−1^, assigned to N–H groups, was observed in control and NaCl leaves, with higher absorbance in the latter.

A sharp peak at 1386 cm^−1^, corresponding to C–H symmetrical deformation, displayed the highest absorbance under NaCl, followed by NaCl + Si, which approached control levels. At 1241 cm^−1^, a peak related to P=O stretching showed the highest absorbance in NaCl‐treated leaves, with NaCl + Si exhibiting values close to control.

The peak at 1042 cm^−1^, assigned to C–O vibrations, was prominent in all treatments, with similar absorbance across NaCl, NaCl + Si, and control, indicating no Si effect on this group. Additional peaks at 892, 825, 594, and 523 cm^−1^ corresponded to out‐of‐plane C–H deformation, C–O–O–C stretching, O–H deformation, and in‐plane deformation of phenolic rings, respectively. Peaks at 825 and 594 cm^−1^ were absent in NaCl + Si leaves but present with high absorbance under NaCl. The 892 cm^−1^ peak was similar in NaCl and NaCl + Si treatments, whereas the 523 cm^−1^ band showed slight attenuation with Si addition compared to NaCl, indicating a modest reduction in phenolic ring accumulation.

### Correlation analysis

Correlation analyses between the applied treatments (NaCl and NaCl + Si) and the studied parameters were conducted (Table 2).

**Table 2:** Pearson correlation matrix of the measured variables under sodium chloride (NaCl) and silicon (Si) supplementation (NaCl + Si) treatments

Scale	Variables	NaCl	NaCl + Si
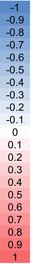	Root dry weight	−0.737 ***	0.737 ***
	Shoot dry weight	−0.677 **	0.677 **
	Whole plant dry weight	−0.775 ***	0.775 ***
	Root length	0.791 **	−0.791 **
	Shoot length	−0.836 ***	0.836 ***
	Leaf number	−0.516 *	0.516 *
	Tiller number	0.886 ***	−0.886 ***
	Salt tolerance index	−0.775 ***	0.775 ***
	Chlorophyll *a*	−0.765 **	0.765 **
	Chlorophyll *b*	−0.769 **	0.769 **
	Total chlorophyll	−0.772 **	0.772 **
	Carotenoid	−0.886 **	0.886 **
	Root Si content	−0.990 ***	0.990 ***
	Leaf Si content	−0.998 ***	0.998 ***
	Silicon translocation factor	−0.995 ***	0.995 ***
	Root soluble sugar content	−0.696 *	0.696 *
	Leaf soluble sugar content	−0.715 **	0.715 **
	Root proline content	−0.990 ***	0.990 ***
	Leaf proline content	−0.691 *	0.691 *
	Leaf malondialdehyde content	0.789 *	−0.789 *
	Palmitic acid (C16:0)	0.979 ***	−0.979 ***
	Palmitoleic acid (C16:1)	0.997 ***	−0.997 ***
	Stearic acid (C18:0)	0.841 *	−0.841 *
	Oleic acid (C18:1)	0.699 *ns*	−0.699 *ns*
	Linoleic acid (C18:2)	−0.946 **	0.946 **
	Linolenic acid (C18:3)	−0.990 ***	0.990 ***
	Double bond index (DBI)	−0.999 ***	0.999 ***
	Saturated fatty acid (SFA)	0.977 ***	−0.977 ***
	Monounsaturated fatty acid (MUFA)	0.982 ***	−0.982 ***
	Polyunsaturated fatty acid (PUFA)	−0.999 ***	0.999 ***
	Unsaturated fatty acid (UFA)	−0.987 **	0.987 **
	Unsaturated fatty acid/saturated fatty acid ratio (UFA/SFA)	−0.999 ***	0.999 ***

*Note*: Treatments were treated as qualitative variables, and physiological and biochemical parameters as quantitative variables. Variables were centred on their means and normalized to have a standard deviation of 1. Negative correlations are shown in blue, positive correlations in red; intensity reflects correlation strength. Bold values with asterisks indicate statistically significant correlations (*P < 0.05; **P < 0.01; ***P < 0.001). ns, not significant (*P* ≥ 0.05).

Under salt stress (200 mmol L^−1^ NaCl), multiple negative correlations were observed, including root, shoot, and total plant dry weight, shoot length, leaf number, STI, Chl. *a*, Chl. *b*, total chlorophyll, Car. content, and root and leaf soluble sugar and proline contents. In addition, C18:2, C18:3, FA DBI, PUFA, total UFA, and the UFA/SFA ratio also correlated negatively with NaCl treatment. Conversely, NaCl treatment was positively correlated with increased root length, tiller number, leaf MDA content, C16:0, C16:1, C18:1, as well as total SFA and MUFA.

With Si supplementation, several positive correlations were recorded, highlighting its beneficial effects in promoting improvements across multiple agronomic, physiological, and biochemical parameters. These included increases in root, shoot, and total plant dry weight, shoot length, leaf number, photosynthetic pigment levels, soluble sugar and proline contents in both roots and leaves, as well as PUFA and total UFA contents. The UFA/SFA ratio and DBI also showed significant positive correlations with Si supply, reflecting a decrease in SFA and an increase in UFA, respectively. Furthermore, MDA content was negatively correlated with Si supplementation, consistent with the observed reduction in oxidative damage in *S. bicolor* leaves (Table 2 and Fig. 10).

**Figure 10:**
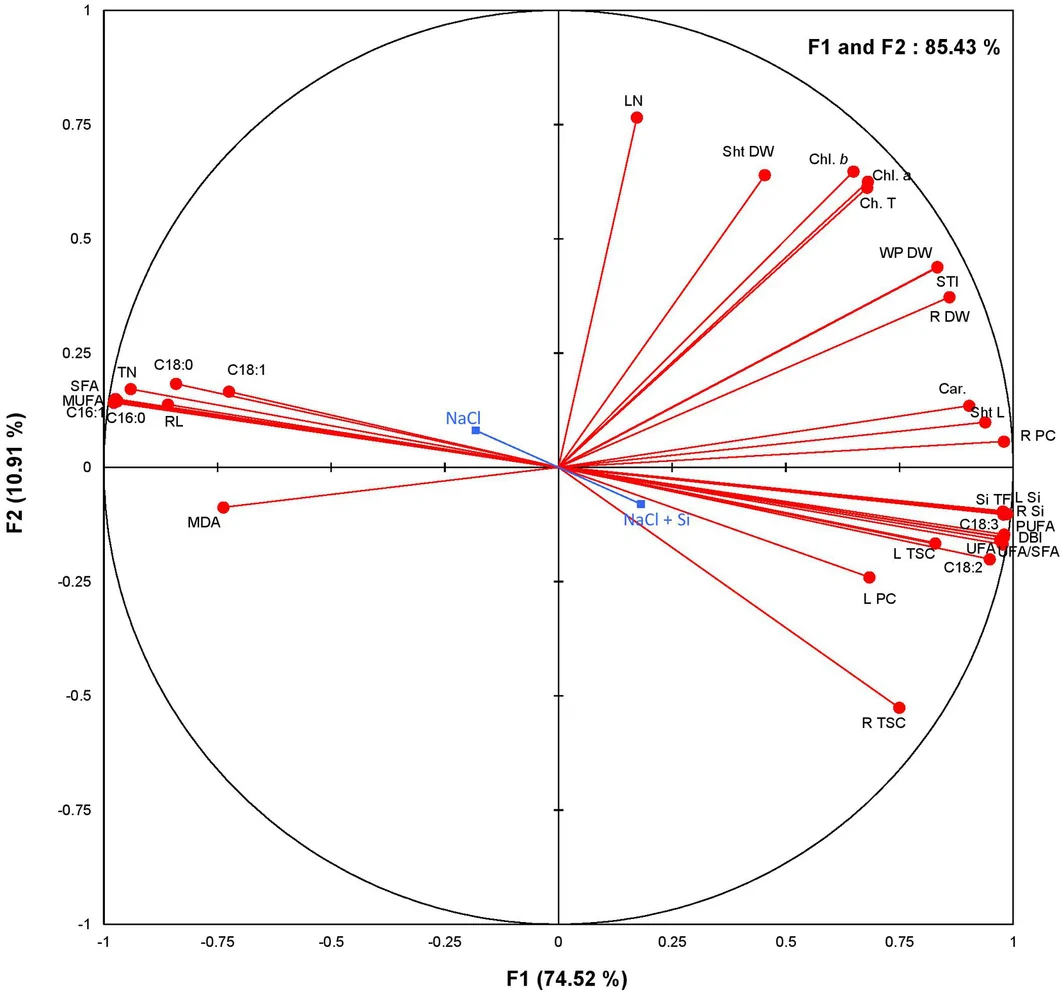
Principal component analysis (PCA) of the continuous measured variables. In the F1–F2 projection, acute angles between variable vectors indicate positive associations, obtuse angles indicate negative associations, and angles near 90° indicate weak associations; these interpretations are approximate because the first two components do not capture all variance. The PCA compares NaCl and NaCl + Si treatments and explains 85.43% of the total variance. Abbreviations: R DW, root dry weight; Sht DW, shoot dry weight; WP DW, whole plant dry weight; STI, salinity tolerance index; RL, root length; Sht L, shoot length; LN, leaf number; TN, tiller number; Chl. *a*, chlorophyll *a*; Chl. *b*, chlorophyll *b*; Chl. T, total chlorophyll; R TSC, root total sugar content; L TSC, leaf total sugar content; R PC, root proline content; L PC, leaf proline content; R Si, root silicon content; L Si, leaf silicon content; Si TF, silicon translocation factor; L MDA, leaf malondialdehyde content; C16:0, palmitic acid; C16:1, palmitoleic acid; C18:0, stearic acid; C18:1, oleic acid; C18:2, linoleic acid; C18:3, linolenic acid; DBI, double bond index; SFA, saturated fatty acid; MUFA, monounsaturated fatty acid; PUFA, polyunsaturated fatty acid; UFA, unsaturated fatty acid; UFA/SFA, unsaturated fatty acid/saturated fatty acid ratio.

## DISCUSSION

The negative impact of salt stress on plant productivity is a widely reported phenomenon across several species, including sorghum.^47^, ^48^, ^49^ In this study, salt‐stressed *S. bicolor* plants exhibited a significant reduction in growth, evident from decreased root, shoot, and total plant dry weight. Additionally, shoot length decreased under salt stress, whereas root length and tiller number increased, with no significant change in leaf number. In fact, plants exposed to saline environments face the lower water potential of the growth medium as compared to that of *S. bicolor*, resulting in osmotic stress.^3^ This difference in water potential creates disruptions in ionic homeostasis and also water deficit, which often leads to stomatal closure and reduced photosynthetic efficiency, ultimately decreasing crop yield.^50^, ^51^ As a consequence, the observed increase in root length together with the decrease in shoot length illustrates an adaptive response of salt‐stressed *S. bicolor* to search for nutrients and water, where more energy is allocated to the roots to increase their surface to face osmotic and water stress.^51^ Moreover, tiller number is a key trait contributing to crop yield, as it determines panicle numbers and affects grain production.^52^ Thus, the increase in tiller numbers under salt stress may serve as a tolerance mechanism, enabling plants to compensate for reduced growth through reproductive adjustments. Nevertheless, Si supplementation restored root, shoot, and total plant dry weight, as well as root and shoot length, to control levels. Additionally, the tiller number was also restored, while the leaf number increased beyond control values, highlighting Si's beneficial effect on yield‐improving. This effect has been primarily attributed to reduced Na^+^ toxicity, maintenance of tissue hydration, enhancement of photosynthetic activity, improved osmotic regulation, and up‐regulation of the enzymatic antioxidant defence system.^53^ Further, these beneficial effects have been widely documented, both via foliar application of Si^54^, ^55^, ^56^ as well as through root‐mediated medium supply of Si in Si‐accumulating species.^25^, ^30^, ^57^, ^58^ Therefore, assessing Si's absorption via roots is essential to verify its accumulation in *S. bicolor* tissues. Indeed, *S. bicolor* plants exposed to salt stress and supplemented with Si (0.5 mmol L^−1^ Na_2_SiO_3_) exhibited significant Si accumulation, primarily in leaves, indicating uptake via roots and subsequent translocation to leaves following the transpiration flow and its deposition in leaf epidermal cells.^36^ Photosynthetic pigments are essential components of the photosynthetic machinery, integral to the structure and function of PSII and PSI.^59^, ^60^ Under salt stress, *S. bicolor* leaves exhibited a significant decline in Chl. *a*, total chlorophyll (Chl. *a* + Chl. *b*), and Car. content. This reduction contributes to decreased plant growth and biomass production, given the central role of chlorophyll in primary metabolism. Similar results have also been reported in sorghum, tomato, and wheat.^47^, ^61^, ^62^ This decrease may result from either reduced biosynthesis or increased degradation mediated by stress‐induced chlorophyllase activity.^63^ Results also showed that Chl. *b* content remained stable under salt stress. According to Voitsekhovskaja and Tyutereva,^64^ PSII and PSI reaction centres contain only Chl. *a*, whereas light‐harvesting antenna complexes (LHCI and LHCII) include both Chl. *a* and Chl. *b*, with Chl. *b* contributing approximately 22% of total chlorophyll in LHCI, 43% in LHCII, and 31–46% in minor antenna proteins. Thus, the stability of Chl. *b* under salt stress is crucial for maintaining light‐harvesting efficiency. More importantly, Si supplementation increased Chl. *a*, total chlorophyll, and Car. and even Chl. *b* contents, which contributes to the improved growth of *S. bicolor* under NaCl + Si conditions. In fact, Si has been shown to exhibit a protective effect on chlorophyll from degradation and promotes leaf area expansion, enhancing light capture and photosynthetic performance.^53^ The supply of Si to the culture medium was also shown to enhance the accumulation of soluble sugars and proline in roots and leaves of salt‐stressed *S. bicolor*. Plants adapt to osmotic stress by synthesising and accumulating compatible solutes like proline and soluble sugars to maintain osmotic balance between cells.^65^ The regulation of osmotic adjustment by Si in salt‐stressed plants has been widely studied. While some studies have reported findings consistent with the present results,^27^, ^66^ others have reported the opposite, showing that Si can decrease proline content,^55^, ^67^, ^68^ highlighting the variable role of Si in ameliorating salt stress depending on species tolerance, stress severity, and growth conditions. In the present study, the stimulatory effect of Si on proline content may play a central role in the salt tolerance of *S. bicolor*. In fact, beyond its function in osmotic adjustment, proline also acts as a stress signalling molecule, a detoxifying ROS, a cytosolic pH buffer, and a stabiliser of molecular structures and membranes, including PSII, thereby promoting salt tolerance in *S. bicolor*.^69^ Furthermore, as reported by Abdelaal *et al*.,^26^ increased proline also contributes to the protection of chlorophyll from degradation, which may explain the observed increase in chlorophyll content with Si supply, promoting photosynthesis. Based on this, we can suggest that Si exhibits a linked protective effect on plant metabolism, which confers better tolerance against salinity, via promoting osmotic adjustment together with chlorophyll protection.

It is evident that reduced plant growth under salt stress is strongly associated with inhibited photosynthesis.^70^ In sensitive species, salt stress commonly reduces PSII and PSI yields, inhibiting photosynthetic activity and ultimately reducing plant productivity.^71^ However, results of this study revealed a contrasting response of *S. bicolor* photosystems to salt stress. Whereas Y(II) and ETR(II) showed an increase under 200 mmol L^−1^ NaCl compared to the control, Y(I) and ETR(I) decreased, reflecting inhibition of PSI. More interestingly, the observed increase of Y(II) and ETR(II) under salt stress was more evident with Si supply, while the decrease of Y(I) and ETR(I) was suppressed and restored to control level. In fact, PSII plays a critical role in photosynthesis, initiating the first photosynthetic chemical reaction via the light‐driven water‐plastoquinone oxidoreductase reaction, in which electrons are transferred from water to plastoquinone.^72^ When the photosynthetic electron transport chain is over‐reduced, excessive excitation energy of chlorophylls can be transferred to oxygen, forming singlet oxygen (_1_O^2^) and subsequent ROS.^73^ The higher Y(II) and apparent ETR(II) values under NaCl and NaCl + Si indicate greater PSII photochemical utilisation under the measurement conditions. However, these parameters alone do not demonstrate a compensatory response aimed at preventing ROS production because ROS generation, leaf absorptance, excitation partitioning, and alternative electron sinks were not measured. Previous research has also demonstrated that an elevated proline and sugar content, as revealed previously with Si addition, may also contribute to PSII photoprotection against osmotic and oxidative stress.^74^, ^75^, ^76^ This observation illustrates Si's role in improving PSII photochemical efficiency.

According to Klughammer and Schreiber,^42^ Y(II) represents the fraction of light energy conversion used photochemically by PSII. The remaining energy is dissipated via non‐photochemical processes, divided into Y(NPQ), the quantum yield of regulated non‐photochemical energy quenching, which dissipates excess energy as heat, and Y(NO), representing unregulated non‐photochemical energy dissipation. In this study, the increase in Y(II) and ETR(II) under salt stress was accompanied by an increase in Y(NPQ), which confirms the rise in excitation energy of electrons that is not used in the photochemical reaction and thus its dissipation as heat. However, a decrease in Y(NO) was observed, indicating reduced unregulated energy dissipation. As noted by Qu *et al*.,^77^ a higher Y(NPQ) curve indicates that photon flux exceeds the photosynthetic pigment capacity in LHCII, prompting energy dissipation as heat to prevent _1_O^2^ formation. The thylakoid ΔpH gradient is essential for cytochrome b6f‐dependent regulation of linear electron transport, protecting PSI from photooxidative damage.^78^ The increase in Y(NPQ) indicates a greater fraction of absorbed energy allocated to regulated non‐photochemical dissipation. Silicon addition reduced Y(NPQ) toward control values and further decreased Y(NO), while Y(II) increased. Because the proton‐motive force and lumen pH were not measured, these changes cannot be interpreted as direct evidence of altered lumen acidification. They indicate redistribution of absorbed energy among PSII photochemistry, regulated dissipation, and non‐regulated dissipation.

Further results showed that salt stress also increased qP and qL compared to the control, consistent with observations in Moringa leaves under salinity.^79^ The qP and qL values represent, respectively, the proportion of photons absorbed by P680 utilized in open reaction centres, and the proportion of open PSII centres. The increase in qP and qL indicates more open PSII reaction centres under salt stress, explaining the stimulation of Y(II). This increase was more pronounced with Si supplementation, aligned with the observed increase in Y(II) and ETR (II) and the decrease in Y(NPQ) and Y(NO), further demonstrating Si's role in improving PSII photochemical efficiency.

Comparing the photochemical activity of PSII and PSI, the over‐stimulation of PSII under salt stress relative to PSI may, nevertheless, disrupt the overall photosynthetic balance, reducing photosynthetic efficiency.^78^ For PSI, the decrease in Y(I) and ETR(I) under salt stress was also accompanied by an increase in Y(ND) and a decrease in Y(NA). Y(ND) reflects non‐photochemical energy dissipation in PSI which is due to electron donor‐side limitation, representing the oxidized fraction of P700^+^. Thus, a salt stress‐induced increase in Y(ND) indicates higher P700^+^ levels, with PSI being in a constantly oxidized state due to a lack of electron transfer from the donor‐side. This result was supported by the increase of P700ox. The increase in Y(ND), together with greater P700 oxidation, is consistent with stronger donor‐side limitation of PSI, whereas the decrease in Y(NA) indicates weaker acceptor‐side limitation. This pattern is compatible with restricted electron supply to PSI but does not identify the location or cause of that restriction or directly quantify electron flow from PSII to PSI.^80^ In fact, as stated by Baker *et al*.,^81^ across most of the PAR intensities, the excitation of PSII has been proven to usually exceed that of PSI, which often leads, specifically under stress conditions, to an overreduction state of electron acceptors of PSII and thus to an overreduction of the electron transfer chain and photooxidative damage to PSI by ROS. This is mainly attributable to the fact that the oxidation of the plastoquinone pool is the slowest step in the photosynthetic electron transport chain and also the amount of cytochrome b6f is normally lower than those of PSII and PSI in plant leaves.^82^ As a result, this damage is mostly minimized by the slower consumption of adenosine triphosphate (ATP) by the Calvin Benson cycle, ensuring in return that proton (H^+^) influx exceeds its efflux from the lumen, which creates a ΔpH that triggers a reduced electron transfer to PSI throughout the restriction of the cytochrome b6f complex and thus the evident oxidation of PSI. This observation explains the rise of energy dissipation in PSII under its regulated form (Y(NPQ)) and also in PSI due to donor side limitation (Y(ND)).^83^

Interestingly, Si supply restored electron transfer to PSI, as evident with the increase in ETR(I), alongside a decreased Y(ND) and P700 oxidation, reflecting improved electron flow from PSII to PSI. Taken together, the addition of Si promoted PSII activity (increase in Y(II) and ETR(II)) concomitantly with lowering the need for energy dissipation in PSII (decrease Y(NPQ) and Y(NO)), as a consequence of unblocking the electron transfer chain, specifically the donor side of PSI (decreased Y(ND) and P700ox), leading to a recovered PSI photochemical activity (increased Y(I) and ETR(I)). This finding significantly demonstrates the mechanistic role of Si in protecting the photochemical phase of photosynthesis in salt‐stressed *S. bicolor*, via selectively restoring PSII and PSI activities and rebalancing the functional coordination in between.

In conjunction with photosynthetic efficiency, membrane stability is also a key indicator of plants' physiological stability under salt stress. In fact, membranes are primarily composed of lipids with a hydrophilic polar head and hydrophobic tails consisting of FAs. FAs may be SFAs with single carbon–carbon bonds or UFAs with one or more double bonds, including MUFAs and PUFAs.^84^ Aldehydes, such as MDA, are derived from lipid peroxides (L5OOHs) resulting from ROS‐mediated oxidation, particularly of PUFA, which makes membranes susceptible to oxidation.^85^, ^86^ Results of this study showed that MDA levels increased in *S. bicolor* leaves under salt stress, whereas Si supplementation attenuated its accumulation. This observation suggests reduced oxidative damage to FAs, especially to PUFAs. Similar protective effects of Si on membrane lipids were also reported in sorghum, sunflower, and basil.^55^, ^87^ To corroborate this finding, leaf FA analyses were also conducted as a complementary approach.

In *S. bicolor* leaves, results showed a qualitative similarity and quantitative modifications of FA under the different studied treatments. Under salt stress, palmitoleic acid (C16:1) showed a significant increase and became the most abundant FA compared to control, followed by C16:0, C18:3, C18:1, C18:2, and C18:0. Quantitatively, while most FAs increased under salt stress, only C18:2 and C18:3 showed a significant decrease, reducing the overall PUFA content. Similar decreases in C18:3 were also reported in *Sulla carnosa* and *Sulla coronaria* under salinity.^88^ In fact, C18 species are particularly prone to ROS‐induced lipid peroxidation, which might explain the decrease in C18:2 and C18:3 contents associated with the observed MDA increase.^89^ Salt stress also disrupts enzymatic activities and FA transport, leading to increased C18:1 and decreased C18:2/C18:3.^90^, ^91^ As a result, the reduced PUFA content affected the DBI, UFA/SFA ratio, and total UFA, thereby impairing membrane fluidity and photosynthetic function.^10^, ^14^, ^92^, ^93^ MUFAs (C16:1, C18:1) and SFAs (C16:0, C18:0) were also increased with salt stress, consistent with the adaptive responses reported in other species.^7^, ^94^, ^95^, ^96^, ^97^ Such changes reduce membrane permeability to salt ions.^15^ Notably, with Si supply to salt‐stressed *S. bicolor* plants, all modifications induced by salt stress were mitigated as follows: while MUFA levels significantly declined, PUFA showed a significant increase, resulting in a rise in total UFA content, consistent with the control. Consequently, the DBI was restored, reflecting the recovery of FA unsaturation. Additionally, SFAs decreased, leading to a rise in the UFA/SFA ratio. Similar results were also reported in *Sesamum indicum* grown under semi‐arid conditions, showing a suppression of C16:0 and C18:0 accumulation under salt stress following Si treatment.^98^

Although few studies have explored Si's protective role in FA metabolism, Jam *et al*.^56^ linked Si's role in improving FA quality under salt stress to enhanced uptake of nutrients essential for FA biosynthesis, mediated by improved root growth and increased photosynthetic assimilation. Erfani‐Moghadam *et al*.^99^ further reported that Si protects FA against oxidative stress, which can promote FA biosynthesis and reduce their degradation. Nevertheless, results of this study demonstrate Si's protective role in FA metabolism and membrane integrity, mainly through preserving levels of C18:2 and C18:3, thereby suppressing membrane lipid peroxidation, as reflected by the decrease seen in MDA accumulation.

For a better understanding of Si's role against salt stress and possibly FA metabolism, FTIR analysis was also employed to assess its potential involvement in protecting chemical structures and their relative contents in *S. bicolor* leaf samples. FTIR spectra identified 12 peaks, each representing absorbance at a specific wavenumber, demonstrating distinct differences among the three studied treatments. A broad peak at ~3296 cm^−1^ was observed in all spectra, indicative of O–H stretching vibrations (hydroxyl groups).^100^, ^101^, ^102^ However, differences in absorbance among treatments suggest a higher OH content in salt‐treated leaves, reflecting the stimulation of components such as phenolic compounds, major contributors to antioxidant activity, as an adaptive response to salt stress.^103^ These results are consistent with spectra reported by Ricci *et al*.^104^ in sorghum extracts, indicative of phenolic and tannin compounds. Comparison of all spectra indicated that Si reduced OH group accumulation, bringing it closer to control. Additional shared peaks were also revealed at 2916 and 2862 cm^−1^ corresponding to C–H stretching vibrations of methylene (CH_2_) and methyl (CH_3_) groups, mainly in aliphatic chains, including chlorophylls, Car., and also FAs.^102^, ^103^, ^105^, ^106^, ^107^ In a similar manner, NaCl‐treated samples exhibited the highest absorbance, indicating elevated CH content, whereas Si addition produced an overlapping band with NaCl, suggesting minimal effect of Si on C–H accumulation. Further, a band at ~1619 cm^−1^ corresponded to C=C stretching vibrations, attributed to olefins, such as UFA was also revealed.^102^, ^103^, ^108^, ^109^ Consistently, NaCl treatment showed the highest absorbance, followed by NaCl + Si with intermediate values closer to the control, highlighting Si's role in attenuating C=C accumulation. This result is consistent with the observed decrease in MUFA upon Si addition, which further confirms Si's protective role at the chemical level.

A weak band at ~1525 cm^−1^ was assigned to N–H bending vibrations, associated with chlorophylls and Car.^105^ This band was present only in control and NaCl samples, with NaCl exhibiting higher N–H content. Its absence in NaCl + Si samples suggests Si's influence on specific functional groups, possibly via interaction with N–H.^110^, ^111^ Such results strongly suggest Si's potential implication in modifying the molecule's chemical structure.

In addition, a sharp peak at ~1386 cm^−1^, corresponding to symmetric C–H bending vibrations attributed to saturated aliphatic chains, mainly SFAs.^102^, ^108^, ^112^ Thus, NaCl‐treated leaves showed the highest absorbance, which is consisting with the increase in SFA previously reported, whereas Si addition attenuated this peak, aligning it closely with the control.

Moreover, a peak at 1241 cm^−1^ was ascribed to P=O asymmetric stretching in phospholipids and polyphosphates.^112^, ^113^, ^114^ Similarly, NaCl‐treated samples exhibited higher P=O content, while NaCl + Si samples displayed levels closer to the control, further highlighting Si's mitigation of salinity‐induced changes.

The peak at ~1042 cm^−1^, which showed the highest absorbance across all studied treatments, was associated with C–O bonds (C–OH, C–O–C), characteristic of carbohydrates such as cellulose.^106^, ^115^, ^116^ However, NaCl and NaCl + Si samples showed similar absorbance, indicating minimal Si effect.

Additional peaks at ~892, ~825, ~594, and ~523 cm^−1^ were also observed. The 892 cm^−1^ peak reflected alkenes of out‐of‐plane C–H bending in carbohydrates, or C=CH_2_ olefinic bonds^102^, ^117^ with overlapping bands of NaCl and NaCl + Si samples. The 825 cm^−1^ peak corresponded to C–O–O–C stretching in ethers, and the 594 cm^−1^ peak to out‐of‐plane OH bending. Both peaks were only observed in control and NaCl leaf samples, and were absent in NaCl + Si. This present observation further supports Si's role in modifying secondary structures of molecules. Lastly, the 523 cm^−1^ peak, attributed to in‐plane deformation of phenolic rings,^104^ showed partial attenuation in Si‐treated samples compared to NaCl alone.

In combination, FTIR analysis revealed absorbance variations of some functional groups' content that align with the observed fluctuations in FA contents in response to Si addition (CH_2_, CH_3_, C=C and C–H) and demonstrated that Si also has a significant impact on the structural level of several cellular metabolites. The absence of some specific peaks and modifications in absorbance underscores Si's dynamic role in modulating molecular architecture in *S. bicolor* leaves.

## CONCLUSION

In summary, results of this study proved that Si effectively enhances *S. bicolor* growth, as reflected by increased biomass production and plant size under salt stress. Physiologically, Si positively influenced Chl. *a*, Chl. *b*, total chlorophyll, and Car. contents. Silicon supplementation also improved osmotic adjustment, as evidenced by increased proline and soluble sugar content, enhancing plant tolerance to salinity. Specifically, Si selectively enhanced PSII (further stimulated Y(II) and ETR(II)) and PSI activities (restored Y(I) and ETR(I)), and regained photochemical balance of the photosynthetic electron transport chain, mainly through modulating energy dissipation processes in PSII (decreased Y(NPQ) and Y(NO)) and also in PSI (suppression of donor side limitation Y(ND)), improving as a result electron transport between PSII and PSI. Silicon also exerted a protective role in preserving membrane stability, mainly by reducing lipid peroxidation predominantly shown in reduced MDA content and increased levels of C18:2 and C18:3. Additionally, FTIR results supported results of Si's protective effect on FA metabolism against salt‐induced alterations and also in preserving the secondary structure of *S. bicolor* molecules (Fig. 11).

**Figure 11:**
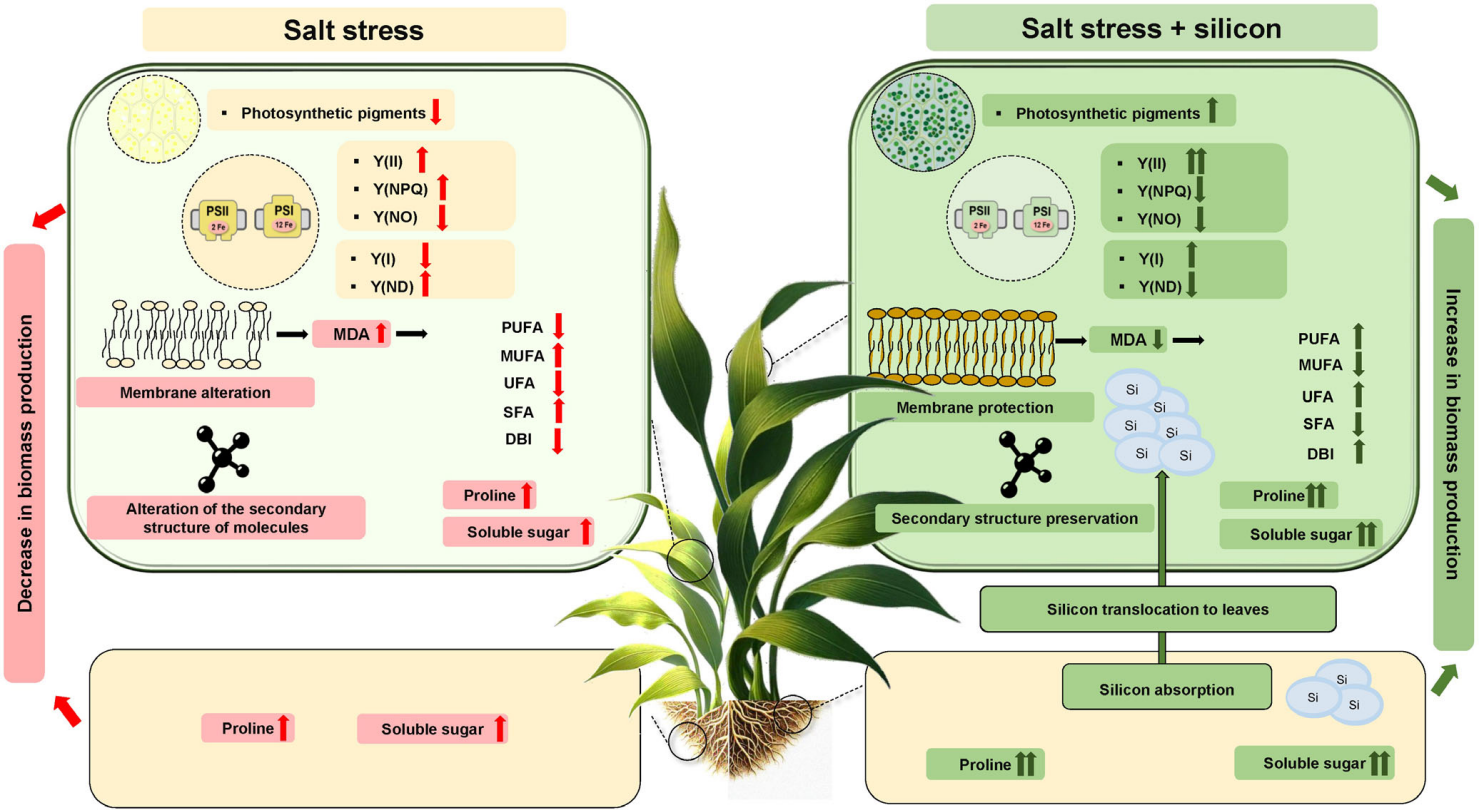
Schematic representation of the physiological effects of salinity stress and silicon (Si) supplementation on *Sorghum bicolor*. Abbreviations: Y(II), quantum yield of photochemical energy conversion in photosystem II (PSII); Y(NPQ), quantum yield of regulated non‐photochemical energy dissipation in PSII; Y(NO), quantum yield of non‐regulated non‐photochemical energy dissipation in PSII; ETR(II), electron transfer rate in PSII; qL, proportion of open PSII centres; qP, fraction of photons absorbed and used by open reaction centres; Y(I), quantum yield of photochemical energy conversion in photosystem I (PSI); Y(NA), quantum yield of non‐photochemical energy dissipation in PSI limited by the acceptor side; Y(ND), quantum yield of non‐photochemical energy dissipation in PSI limited by the donor side; ETR(I), electron transfer rate in PSI; P700ox, oxidation of PSI; MDA, malondialdehyde; PUFA, polyunsaturated fatty acid; MUFA, monounsaturated fatty acid; UFA, unsaturated fatty acid; SFA, saturated fatty acid; DBI, double bond index.

Overall, this study revealed the pivotal role of Si in enhancing crop yield and tolerance to salt stress, majorly acting as a modulator and protector of photosynthesis, membrane integrity, FA metabolism, and molecular structure stability from salt‐induced damage.

## FUNDING INFORMATION

This research was financially supported by the Tunisian Ministry of Higher Education and Scientific Research under grant number LR15CBBC02. Additional support was provided by the PHC Maghreb 2019 network (Partenariat Hubert Curien Maghreb 2019, project number 41482RL‐19MAG41, ‘PROSPECT’ Project), and the Young Researchers Encouragement Programme (Programme d'Encouragement des Jeunes Chercheurs, project number 21PEJCD4P1, led by Walid ZORRIG (‘Si‐Grow’ Project)). Furthermore, this work was conducted under the MOBIDOC scheme, funded by the European Union through the SWAFY Project and managed by the ANPR (Agence Nationale de la Promotion de la Recherche scientifique), supporting the postdoctoral research of Dr Rabaa Hidri (‘Si‐Green’ Project). The final phases of this research work were additionally supported by the PHC‐UTIQUE 2025 network (Partenariat Hubert Curien UTIQUE 2025, project number 25G0905, ‘SiCropSave’ Project).

## CONFLICT OF INTEREST

The authors declare no known conflicts of interest and confirm that no external financial support influenced the outcomes of this work.

## ETHICS STATEMENT

All authors adhered to the ethical standards relevant to this study.

## CONSENT FOR PUBLICATION

All authors consented to the publication of this study.

## AUTHOR CONTRIBUTIONS

Chedly Abdelly and Walid Zorrig conceptualized the study and proposed the research theme. Farah Bounaouara, Rabaa Hidri, Mohammed Falouti, Ahmed Hichem Hamzaoui, Issam Nouairi, Nabil Ben Youssef, Cécile Cabassa, Arnould Savouré, Walid Zorrig, and Inès Slama contributed to the preparation of materials, data collection, and analysis. Salma Nait Mohamed provided technical expertise for gas chromatography‐flame ionization detector analyses of fatty acids. Farah Bounaouara drafted the initial manuscript, and Walid Zorrig, Inès Slama, Rabaa Hidri, and Mokded Rabhi provided constructive feedback on earlier drafts. All authors reviewed and approved the final manuscript.

## Data Availability

The data that support the findings of this study are available from the corresponding author upon reasonable request.
